# Diffuse Gallbladder Adenomyomatosis in a Child

**DOI:** 10.7759/cureus.15555

**Published:** 2021-06-09

**Authors:** Hong Duc Pham, Minh Xuan Ngo, Thu Ha Dang

**Affiliations:** 1 Radiology, Hanoi Medical University, Hanoi, VNM; 2 Pediatrics, Pham Ngoc Thach University of Medicine, Ho Chi Minh City, VNM

**Keywords:** diffuse adenomyomatosis, gallbladder, ultrasonography, magnetic resonance imaging, children

## Abstract

Adenomyomatosis of the gallbladder is a benign condition, usually occurring in middle age, in which the epithelium of the gallbladder proliferates and the gallbladder wall thickens with the presence of Rokitansky-Aschoff sinuses (RAS). The diffuse form is an unusual subtype of adenomyomatosis. Herein, we describe a 17-year-old female who presented with dull and intermittent pain in the right hypochondriac region for more than a month. Ultrasound followed by magnetic resonance imaging showed marked diffuse gallbladder wall thickening. A gross cholecystectomy specimen showed a diffusely enlarged gallbladder. The final diagnosis of gallbladder adenomyomatosis (GA) was confirmed by the histopathologic appearance of muscular and epithelial hyperplasia, contributing to mural thickening with epithelial invaginations forming the pathognomonic intramural diverticula known as the RAS. This case highlights that the diffuse form of GA is uncommon and is often accompanied by chronic inflammation, sometimes requiring differential diagnosis from gallbladder malignancies, especially when there is no image showing a “comet tail” of cholesterol crystals in the wall.

## Introduction

Gallbladder adenomyomatosis (GA) is a benign condition of the gallbladder wall, often asymptomatic and of unknown etiology [1]. It is characterized by mucosal proliferation associated with hyperplasia of the muscularis propria. There are “mucosal pouches” ingrained into the muscular layer that make up the Rokitansky-Aschoff sinuses (RAS), which can be located anywhere but are often at the base of the gallbladder wall and can be like a polyp [2]. The diffuse form is less common and can be confused with acute or chronic cholecystitis, xanthogranulomatosis, and adenocarcinoma [3]. The symptoms are often reported in the right hypochondrial area in most cases. In terms of imaging, ultrasound (US), computerized tomography (CT), and magnetic resonance imaging (MRI) are the most commonly prescribed exams [4]. Herein, we describe a case of diffuse GA that was diagnosed and treated in our hospital.

## Case presentation

A 15-year-old female presented with dull and intermittent pain at the right hypochondriac region for more than one month. She denied nausea, vomiting, fever, jaundice, and change in appetite. Physical examination showed a soft abdomen, with tenderness noted in the right hypochondriac region presenting an atypical Murphy’s sign. Laboratory investigation manifested nonspecific inflammation (leukocytes: 10.9 G/L, neutrophils: 81%, C-reactive protein: 8 mg/L). Other tests for hepatobiliary function were within the normal range.

**Figure 1:**
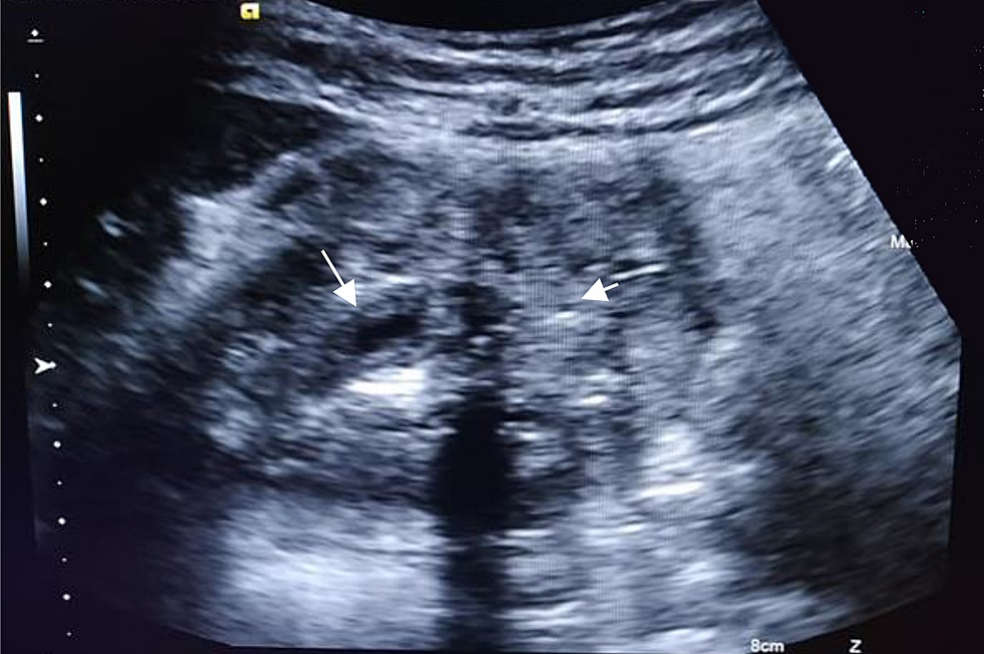
Ultrasonographic longitudinal gallbladder. Diffuse heterogenous wall thickening (short arrow). Note the regular mucosal layer (long arrow) with a small-appearing lumen with evidence of stones.

US revealed an enlarged, heterogeneous gallbladder (measuring about 10 × 6 cm) due to a diffusely thickened wall. The thickened wall was mainly due to hyperechogenic areas interspersed with few echogenic intramural foci, but well-delineated “comet tail” of reverberation artifacts. The gallbladder lumen was very small containing little bile fluid without gallstones, noting that the mucus layer is clearly visible (Figure 1). The outer layer of the wall was well limited, and there was no pericholecystic collection. There was no intra or extrahepatic biliary system dilatation. No enlarged lymph nodes were found. Thus, US suggested the diagnosis of GA, but to exclude gallbladder cancer, the patient was sent for MRI.

**Figure 2:**
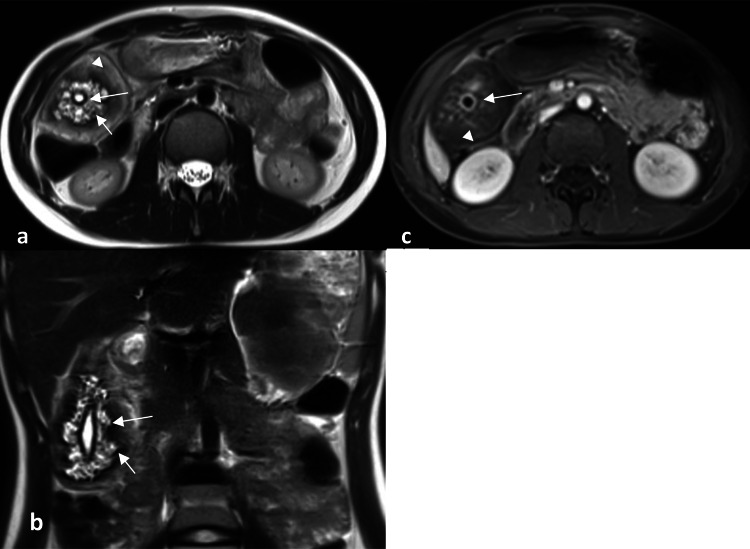
MRI images. MRI, T2W coronal (a), T2W sagittal (b), and T1W axial fat saturation with gadolinium (c). The gallbladder wall has five layers. The innermost zone is hyposignal T2W and enhancement on T1 fat-saturated (long arrow). The second layer has multiple cystic signals of RAS (short arrow). The third layer has hyposignal T2W enveloping the second layer of fibrosis. The fourth zone has hypersignal T2W and hyposignal T1W almost nonenhanced abdominal fat (arrowhead). The fifth outer area is hyposignal on both T1W and T2W of serosa signal. MRI: magnetic resonance imaging; RAS: Rokitansky-Aschoff sinuses; T1W: T1-weighted; T2W: T2-weighted

MRI also showed marked diffuse gallbladder wall thickening. The tiny lumen contained little bile fluid and no gallstones. The muscularis mucosa layer was thin and enhanced regularly. Around this layer, there were multiple variably sized intramural cysts and intraluminal cavities hypersignal on T2-weighted images without void signals of cholesterol crystals. Between the cavities and the serosa were thickened tissue due to fibrosis and fat deposition signals (Figure 2).

**Figure 3:**
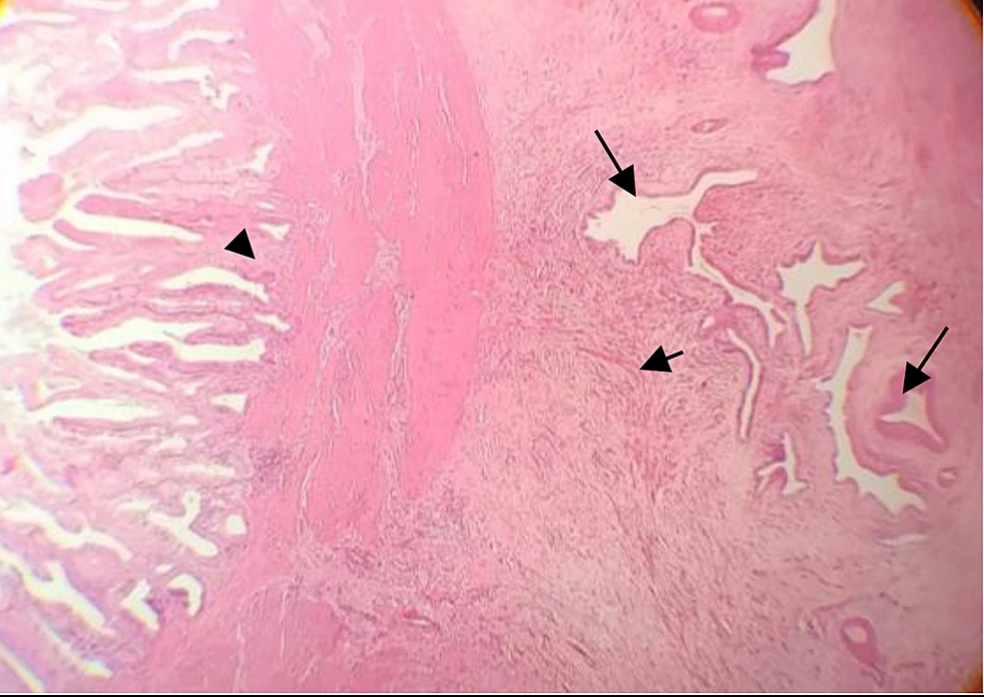
Photomicrograph (original magnification, 4×; H&E stain) showing hyperplastic epithelial column (arrowhead) and several epithelium-lined cystic spaces (long arrows) surrounded by abundant granulation tissue composed of myofibroblasts (short arrow). H&E: hematoxylin and eosin

The patient underwent laparoscopic surgery to remove the gallbladder. A gross cholecystectomy specimen showed a diffusely enlarged gallbladder measuring 11.5 × 6.0 × 5.0 cm. On cut sections, the gallbladder wall was white, fibrous, tough, and firm. The final diagnosis of GA was confirmed by the characteristic histopathologic appearance of muscular and epithelial hyperplasia contributing to mural thickening, with epithelial invaginations forming the pathognomonic intramural diverticula known as RAS (Figure 3).

## Discussion

GA, or adenomyomatous hyperplasia, is a benign hyperplasia of the gallbladder wall and is a relatively common lesion, accounting for 9% of cholecystectomy specimens [5,6]. It is most predominantly found in women in their 50s with the majority presenting with complaints of chronic right upper quadrant pain or with leucocytosis of atypical acute cholecystitis [7].

Dysplasia or carcinoma usually occurs in the epithelial layer of GA, and several authors believe that the cause of cancer development is due to gallstones, chronic inflammation, and dysplastic changes rather than due to the tumor itself. Thus, GAs are not considered malignant lesions [1,3,6].

Adenomyomatosis may involve the gallbladder according to four main patterns: localized, annular, segmental, and diffuse [8]. The segmental is fundus but the annular is in the middle, which is like an hourglass, dividing the lumen gallbladder into the fundal and neck compartment. The annular type can also be considered a subtype of the segmental type [9]. According to Nishimura et al., among 156 GA, the diffuse type only accounts for 1.9%, while the segmental type accounts for 63.5% [9].

Depending on the type, GA can be detected differently on US; the diffuse type can be easily detected with characteristics of a diffuse thick wall with heterogenic tissues. The noticeable sign is intramural echogenic spots with “comet tail” reverberation artifacts. Additionally, twinkling artifacts may also be visible on the color Doppler that project deeply cholesterine crystals into the RAS, which is the diagnostic sonographic hallmark of GA and helps distinguish the disseminated type of gallbladder cancer. However, it should be noted that crystals containing cholesterol of the diffuse type were less common than the segmental type, possibly related to biliary stasis in the fundal compartment [10]. This can be explained by the solid wall of fibrosis that markedly narrows the sinuses on the diffuse GA. Color Doppler usually does not show vascularization in benign chronic inflammatory lesions.

In contrast to US, it is difficult to detect cholesterol crystals on nonenhanced CT scans because they do not contain calcification. Intravenous contrast injection is needed to assess the gallbladder wall thickness. The enhanced, thickened internal muscular layer comprises multiple hypoattenuating cysts, while the external layer contains comprises inflammatory subserosal fibrolipomatous tissue. However, they are nonspecific and should be less indicated if GA is suspected [3].

MRI has recently been described as the best diagnostic modality. Corresponding to the cut surface of the specimen, the wall of GA may show a five-layered mural stratification (Figure 2). The innermost zone corresponds to the lamina muscularis mucosa. The second layer corresponds to a hypertrophic muscular layer with outpouching of the mucosa into the layer, forming the specific pearl necklace sign for GA. The fourth layer corresponds to massive fibrosis with infiltration of several small round cells surrounding the hypertrophic muscular layer. The fifth layer, the subserosal area, is marked with fat deposition. Wall enhancement with gadolinium appears nonspecific for GA and is likely dependent on the number of RAS and subserosal fibrolipomatosis. This fibrous signal wall appears to be a complement to the diagnosis of GA. In some atypical cases, diffusion sequences also help to distinguish the diagnosis from gallbladder cancer [11].

In our case study, the image of the muscular mucosa layer clearly showed a limitation as a form of US. MRI appeared to be a significant additional marker in the differential diagnosis of GA, as all general types, particularly the diffuse type, form polyps, papillomas, adenomas, cystadenomas, as well as gallbladder carcinomas. These lesions alter the epithelial layer and often present as a nodule or mass in the lumen gallbladder.

Another differential diagnosis is xanthogranulomatous cholecystitis, an uncommon variant of chronic cholecystitis, characterized by an inflammatory process containing lipid similar to pyelonephritis. The image also showed that the diffuse thick gallbladder wall with nodules hypoechoic on US and low-attenuation on CT representing abscesses or focal xanthogranulomatous inflammation, more often diagnosed with cancer than GA [3].

Although GA is not considered a risk factor for malignancy, resection is usually indicated in symptomatic cases, in the presence of acute inflammation, in the presence of stones, in secondary inflammation that may lead to dysplasia, and when it is difficult to distinguish GA from other malignancies of the gallbladder [7].

## Conclusions

The diagnosis of GA is usually based on imaging examinations, initially with US. The diffuse form is uncommon and is often accompanied by chronic inflammation. The differential diagnosis is often concerning for a gallbladder malignancy, especially when there is no evidence of the “comet tail” cholesterol crystals in the wall. In these cases, MRI helps determine the diagnosis, mainly based on the presence of RAS. Cholecystectomy is given when patients with GA have symptoms or the diagnosis is unclear.

## References

[1] Bonatti M, Vezzali N, Lombardo F, Ferro F, Zamboni G, Tauber M, Bonatti G (2017). Gallbladder adenomyomatosis: imaging findings, tricks and pitfalls. Insights Imaging.

[2] Pang L, Zhang Y, Wang Y, Kong J (2018). Pathogenesis of gallbladder adenomyomatosis and its relationship with early-stage gallbladder carcinoma: an overview. Braz J Med Biol Res.

[3] Mellnick VM, Menias CO, Sandrasegaran K (2015). Polypoid lesions of the gallbladder: disease spectrum with pathologic correlation. Radiographics.

[4] Caraiani C, Yi D, Petresc B, Dietrich C (2020). Indications for abdominal imaging: when and what to choose?. J Ultrason.

[5] Dilek ON, Karasu S, Dilek FH (2019). Diagnosis and treatment of gallbladder polyps: current perspectives. Euroasian J Hepatogastroenterol.

[6] Mahajan A, Sripathi S (2016). Gallbladder adenomyomatosis mimicking carcinoma: a diagnostic dilemma. J Glob Oncol.

[7] Coulier B, Gielen I, Ramboux A, Van den Broeck S (2016). Symptomatic diffuse adenomyomatosis of the gallbladder with subserosal inflammatory sclerolipomatosis: imaging findings. Diagn Interv Imaging.

[8] Cocco G, Basilico R, Delli Pizzi A (2021). Gallbladder polyps ultrasound: what the sonographer needs to know. J Ultrasound.

[9] Nishimura A, Shirai Y, Hatakeyama K (2004). Segmental adenomyomatosis of the gallbladder predisposes to cholecystolithiasis. J Hepatobiliary Pancreat Surg.

[10] Oishi Tanaka Y, Hori T, Nagata M, Itai Y (2002). Adenomyomatosis with marked subserosal fibrosis and lipomatosis of the gallbladder: mural stratification demonstrated with MR. Magn Reson Med Sci.

[11] Yu MH, Kim YJ, Park HS, Jung SI (2020). Benign gallbladder diseases: imaging techniques and tips for differentiating with malignant gallbladder diseases. World J Gastroenterol.

